# Mutation at the Human D1S80 Minisatellite Locus

**DOI:** 10.1100/2012/917235

**Published:** 2012-05-03

**Authors:** Kuppareddi Balamurugan, Martin L. Tracey, Uwe Heine, George C. Maha, George T. Duncan

**Affiliations:** ^1^School of Criminal Justice, University of Southern Mississippi, 118 College Drive # 5127, Hattiesburg, MS 39406, USA; ^2^Department of Biology, Florida International University, University Park Campus, Miami, FL 33199, USA; ^3^DNA Identification Testing Division, Laboratory Corporation of America, 1440 York Court Extension, Burlington, NC 27215, USA; ^4^Broward County Sheriff's Office, Forensic Laboratory DNA Unit, Fort Lauderdale, FL 33301, USA

## Abstract

Little is known about the general biology of minisatellites. The purpose of this study is to examine repeat mutations from the D1S80 minisatellite locus by sequence analysis to elucidate the mutational process at this locus. This is a highly polymorphic minisatellite locus, located in the subtelomeric region of chromosome 1. We have analyzed 90,000 human germline transmission events and found seven (7) mutations at this locus. The D1S80 alleles of the parentage trio, the child, mother, and the alleged father were sequenced and the origin of the mutation was determined. Using American Association of Blood Banks (AABB) guidelines, we found a male mutation rate of 1.04 × 10^−4^ and a female mutation rate of 5.18 × 10^−5^ with an overall mutation rate of approximately 7.77 × 10^−5^. Also, in this study, we found that the identified mutations are in close proximity to the center of the repeat array rather than at the ends of the repeat array. Several studies have examined the mutational mechanisms of the minisatellites according to infinite allele model (IAM) and the one-step stepwise mutation model (SMM). In this study, we found that this locus fits into the one-step mutation model (SMM) mechanism in six out of seven instances similar to STR loci.

## 1. Introduction

The human genome can be grossly partitioned into three categories: nonrepetitive (single copy sequences), moderately repetitive (families of retroposon-like sequences), and highly repetitive (“classical” satellite) DNA [1]. Minisatellites represent a class of tandem repeats with repeat units ranging from 6 to 100 bp and fall into the class of highly repetitive satellites. Total array sizes range from 0.5 to 30 kb. Minisatellite loci are found throughout the genome and are estimated to number in the thousands with a strong telomeric and strand bias [2]. They are generally clustered in subtelomeric and centromeric regions; however, it has been suggested that minisatellites show no obvious predisposition for accumulation in subtelomeric or centromeric regions but are associated with rates of high recombination and mutation in these regions [2–9].

Studies have shown that minisatellites may mutate by unexpectedly complex conversion-like events [10]. Higher mutational polar bias occurs at the termini at some, if not most minisatellites, as well as microsatellite loci [11]. Minisatellite loci generally are variable near one end of the array or in some cases both ends. It has been suggested that this implies a mutational mechanism driven by cis-acting elements and thus the existence of a localized mutation hotspot at one end of the array involving complex processes of gene conversion [12–14]. One caveat is that the control regions have not been identified and only associated flanking region markers have been found.

**Figure 1:**
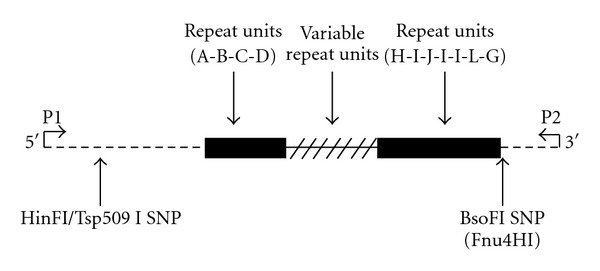
The general structure of the D1S80 locus is that of a monomeric type VNTR consisting of four 5′ repeat units (A-B-C-D) and seven 3′ repeat units (H-I-J-I-I-L-G) that are constant (motifs), with variable number of repeats in between. These two motifs are essentially identical in almost all alleles sequenced in all population groups so far (data not shown). There is a 132 base pair 5′ flanking region which includes the forward PCR primer (P1) and a 32-base pair 3′ flanking region which includes the reverse PCR primer (P2) sequence. The two-flanking restriction site polymorphism, HinfI (G↓ANTC), 58 base pairs from the 5′ end of the primer, and the Fnu4HI (GC↓NGC), the first base after the last repeat are shown by arrows.

Little is known about the general biology of minisatellites [15]. The purposes of the study are (1) to examine the mutational rate and (2) to examine mutations from the D1S80 minisatellite locus by sequence analysis to elucidate the mutational process, as an example of a particular type of minisatellite array. The highly polymorphic minisatellite locus D1S80, (gene location: 1p36.32, GenBank sequence Accession # D28507) was first described by Nakamura in 1989. It is a minisatellite locus composed of short repeat units, typically less than or equal to 16 base pairs per unit [16, 17] (Table 1, Figure 1). It is located in the sub-telomeric region of chromosome 1 (chr1:2,390,716 - 2,391,193) roughly 2.4M bases from the p-telomere [18, 19]. The total range of repeat lengths (approx. 0.2 to 1kb) and the relatively small number of repeats (generally 14 to >40) within the unit make this locus suitable for PCR analysis [15, 20] (Figure 1). The observed heterozygosity has been reported as high as 90.5% and as low as 24% with greater than 27 alleles [21]. In most human populations, 18 and 24 repeat units are the most common and possibly the primordial alleles at this locus [22]. Because of the complexity of the repeat array structure, differences between the alleles become more evident when the arrays of repeat units are divided into common motifs. Six motifs ranging from three to nine repeat units have been identified. Allele frequencies and motifs characterized by the 18 and 24 structural alleles were described earlier [23, 24].

The D1S80 locus was previously used for forensic analysis due to its small size; however, this locus has recently been used as an associative tool in the elucidation of the chromosome 1p36 deletion syndrome which is one of the most common deletion syndromes located near a chromosomal terminus (one in 5000 births) [15, 25].

Recent and past work with respect to this locus has included the use of two single nucleotide polymorphisms (SNPs). These included rs16824398, which is a SNP that involves a HinfI restriction site at a nucleotide 58 bases downstream from the forward primer and another SNP in the 3′ flanking region that involves an Fnu4HI restriction site, at the base next to the last repeat. Interestingly, all 18-repeat alleles to date have been found to be associated with HinfI(+) and Fnu4HI(−) restriction site polymorphisms at the 5′ and 3′ ends, respectively (Figure 1). On the other hand, allele 24 is associated with HinfI(−) and Fnu4HI(+) polymorphism. Of the alleles tested, 98.5% exhibits linkage disequilibrium between two specific SNPs. If an allele is positive for HinfI, then it is negative for Fnu4HI, and if an allele is negative for HinfI, it is then positive for Fnu4HI. There is very strong linkage disequilibrium between the two SNPs [23, 26, 27]. In this study, we report the mutation rate and a nonpolarized mutational mechanism at the D1S80 minisatellite locus.

## 2. Materials and Methods

### 2.1. Sample Collection and Parentage Analysis

Buccal epithelial cell samples from 45,000 trios for paternity analysis were collected in quadruplicate from each individual using cotton-tipped swabs and air dried at ambient temperature. Each paternity short tandem repeat (STR) assay was done using duplicate independently prepared DNA extractions and assayed using proprietary inhouse STR multiplexes. Each multiplex included a shared locus (generally D1S80) with other multiplexes to confirm sample identity. D1S80 was assayed in combination with two to three other STR loci in multiplex reactions. The other STRs were selected to produce lower molecular weight fragments that would not interfere with analysis of the larger D1S80 amplified products. Amplified products were analyzed using nondenaturing high-resolution polyacrylamide gel electrophoresis with silver stain detection. Alleles were manually determined by comparison with allelic ladders containing all common D1S80 alleles. All STR-based parentage testing was performed with full knowledge and consent of the tested individuals or authorized parent/guardian. Appropriate institutional review board approval was obtained for sequence analysis of the samples.

### 2.2. Parentage and Mutation Analysis

To fully understand the implications and delineate the mutational mechanisms that the D1S80 locus undergoes, we examined the number of parent child allele transfers analyzed at this locus for the year 1996 from Laboratory Corporation of America, Burlington, NC. Observed mutations were confirmed as coming from concordant father or mother by analyzing short tandem repeat (STR) data from samples collected from alleged father, mother, and child using Amp*fl*STR Identifiler human identification kit (Applied Biosystems, Foster City, CA). This was essential to confirm that the mutation occurred in the D1S80 locus and was not the result of nonpaternity.

Several factors were used in the elucidation of the origin of the mutation between the alleged father and mother of the child. The first factor was the phase of the SNPs at the 5′- and 3′-flanking sequence of the repeat array. Another factor was a match of the repeat units of the alleles most likely to be associated between father and mother and finally we assumed that the minimal size change was the most probable one.

A mutation rate was then estimated by the number of samples sequenced that contain the mutation and compared to the number of trios sampled that year at Laboratory Corporation of America. Parentage calculations and mutation rate analysis were performed following guidelines put forth by the American Association of Blood Banks (AABB) [28].

### 2.3. DNA Analysis and Sequencing

Total genomic DNA was isolated from the trios that contained the mutation by standard organic extraction with phenol/chloroform/isoamyl alcohol followed by purification and concentration using Amicon Ultracel centrifugal filters (Millipore Corporation, Billerica, MA, USA). Quantitation of total genomic DNA was performed using Quantifiler human DNA quantitation kit (Applied Biosystems, Foster City, CA, USA). Paternity was confirmed by analyzing a set of fifteen short tandem repeat (STR) markers using the Identifiler human identification kit (Applied Biosystems, Foster City, CA, USA) as per manufacturer's recommendations. The samples were run on an automated 310 Genetic Analyzer and analyzed with GeneMapper ID software version 3.2 (Applied Biosystems, Foster City, CA, USA). The D1S80 locus was amplified using primers described by Kasai et al. [16]. Alleles were separated by 5% polyacrylamide gels and stained with ethidium bromide. Individual allelic bands were excised from the gel using disposable razor blades and suspended in 100 *μ*L of Tris-EDTA (TE) buffer overnight. One to five *μ*L of the gel extract was used for amplification of single alleles using the same primers described above. The alleles were sequenced using big dye terminator chemistry and ABI 310 genetic analyzer (Applied Biosystems, Foster City, CA, USA). Homozygous samples or samples that did not yield clean sequence were cloned into pBluescript plasmid vector. The DH5*α* competent cells were transformed using the recombinant vector and the cells were grown in LB/ampicillin agar plates. The resulting colonies were screened for the presence of the right size insert by directly sequencing the plasmid DNA isolated from minipreps of individual colonies. Sequencing of the cloned alleles was performed using big dye terminator chemistry and the sequence was analyzed using Applied Biosystems DNA sequencing analysis software version 5.2.

## 3. Results and Discussion

Paternity calculations were performed using AABB guidelines [28] and a residual likelihood ratio from a low of 180,848 to a high of 190,926,913,150 was observed. Mutations were identified as non-Mendelian inheritance of alleles differing in size in seven (7) out of a total of 45,000 trios (Table 2). Most of the mutations (6/7) involved a change of one repeat unit (Table 2). Although one mutation involved a two-repeat deletion, our observations support the strict step-wise replication slippage mutation (SSM) model according to which the majority of the mutational events at microsatellite loci involve gain or loss of one-repeat unit rather than the complex mutational events as illustrated by other minisatellites. We assumed that the smallest size change was most probable, thus the most parsimonious changes were assumed in our analysis of the mutational events [29, 30]. The phase of the HinfI and Fnu4HI restriction site polymorphisms of all mutated alleles of the children was concordant to the phase of the polymorphisms of the progenitor. Alec Jeffreys, who has examined these mutational events in detail, has stated that replication slippage and unequal crossover events which are intrinsic to the tandem repeat array are not primarily the mechanisms of mutation. Instead, he postulated that germline repeat instability which is regulated by cis-acting elements somewhere near the array may be responsible [31]. In our data, in four trio sets, it was not possible to ascertain which parent was the progenitor of the mutation. In the remaining three trios, we were able to ascertain two mutations to the father and one to the mother. Other studies have emphasized the origin of most mutations from paternal lineage due to more numbers of meiotic events [32–34], but we cannot make this prediction from our data since four of the seven mutations are indeterminate. The advantage of samples like this permits the analysis of true *in vivo *germline events rather than somatic mutational events.

Only two studies are reported that have dealt with the mutational events within this locus [35–37]. The first study [35] reported three mutations involving the loss or gain of three repeats to as much as a loss of six repeats. Of these events, two could be attributed to a maternal origin and the remaining event could not be discerned between mother and father. The data of our study does not support these findings where most mutational events involved one repeat loss or gain. Their conclusion was that paternal events were most prevalent in microsatellite loci, while in minisatellite loci maternal events were most prevalent. The second study dealt with computer simulations and a stepwise mutation model [37]. Jeffreys et al. found that minisatellites do not mutate by replication slippage and concluded that most mutations were exhibited in the male germline [14].

Mutation rates for minisatellites have been estimated to range from 0.5% to greater than 20% per generation in some studies and in another study the rate was reported as 0.53 × 10^−3^ to 1.53 × 10^−3^ [35]. Only one study up to this time has examined the D1S80 locus specifically and found a mutation rate of 3 out of 6153 meioses or 5 × 10^−4^ [35]. We found 7 mutations out of 90,000 meioses (45,000 paternal and 45,000 maternal meioses). This then represented an effective overall mutation rate of approximately 7.77 × 10^−5^. The mutation rate of 7.77 × 10^−5^ represents the overall mutation rate rather than a rate specific to the male or female germline. Only one event could be narrowed down to the mother and two events attributable to the father. In the remaining four trios, we could not choose the progenitor because of the similarities of the maternal and paternal sequences. For the four indeterminate mutations, the AABB prorates them based on known mutation rates [28]. In this case, one adds 2.667 to the males and 1.333 to the female for estimated mutation rates of 1.04 × 10^−4^ (4.667/45,000) and 5.18 × 10^−5^ (2.333/45,000), respectively. Some mutations among the trios might not appear as Mendelian errors in the analysis since the scoring was based on a size difference between alleles (difference in the number of repeats between parent and child), and single base changes would not be detected, and the actual mutation rates could be slightly higher.

Several studies have examined mutational mechanisms according to infinite allele model (IAM) and the one-step stepwise mutation model (SMM). They have found that minisatellites sometimes fit both mechanisms; however, short tandem repeat loci (STR) were most similar to the simulation results under the SMM model of mutation. In this study, we found that this locus fit into the one-step stepwise mutation model (SMM) mechanism in six out of seven instances similar to STR loci [31, 37, 38]. Jeffreys, when using single-molecule polymerase chain reaction (SP-PCR) for elucidation of the minisatellite MS1, found an abundance of mutational events in sperm consisting of the deletion or addition of one-repeat unit [38].

Jeffreys et al. [31] reported polarized mutational events for the three minisatellite loci MS32, MS205, and MS31A. In this study, the authors reported that 93% of mutants lays within 20 repeats of the progenitor allele and 90% of the mutant alleles which had gained repeats showed extreme polarity, with repeat segments being added in the 5′ region of the progenitor allele. This finding is in contrast to our results in this study. All the mutated alleles have deletion or duplication in close proximity to the center of the repeats, while four repeats in the 5′ end and seven repeats in the 3′ end are relatively constant in both the progenitor and the mutated alleles.

In addition to the differences in the repeat regions of the progenitor and mutated alleles, Jeffreys et al. have also found that the polymorphisms flanking the minisatellite repeat array have failed to reveal exchange of flanking markers [31]. This is true with the results obtained from this study that the 5′ HinFI and the 3′ Fnu4HI restriction site polymorphisms are concordant with the progenitor and the mutated alleles. This phenomenon suggests that the 5′- and 3′-flanking regions and at least four repeats on either end of the repeat array are not involved in the mutational process. These differences in the region of the mutation in the repeat array indicate possible different mutational mechanisms in different minisatellite loci.

The data we have reported highlights the importance of a number of questions. (1) Do repeats have functions and do they have an effect on genome structure? (2) Are the allelic clades the result of constraints on mutation? (3) Is selection acting at the D1S80 locus?, and (4) Why is the D1S80 locus conserved among primates (unpublished data) including humans?

## Figures and Tables

**Table 1:** Nucleotide sequences of observed repeat units. The consensus sequence represents the most common nucleotide observed in each position of the repeats. Twenty variations based on the consensus sixteen base repeat unit are tabulated. Each repeat unit is assigned a letter code. Dots (·) represent a match to the consensus sequence as represented by type H repeat unit. Letters represent nucleotide differences when compared to the consensus sequence and correspond to A, G, C, and T nucleotides. A (-) represents a missing nucleotide in the repeat unit A.

Repeat type	Sequence																
Type A	T	C	A	·	C	·	·	·	-	A	·	·	-	·	·	·	
Type B	A	C	A	·	·	·	·	·	·	A	·	·	·	·	·	·	
Type C	·	·	·	·	·	·	·	·	·	·	·	·	A	·	·	·	
Type D	·	·	A	·	·	·	·	·	·	·	·	·	A	·	·	·	
Type E	·	·	A	·	·	·	·	·	·	A	·	·	·	·	·	·	
Type F	·	·	·	·	·	·	·	·	·	A	·	·	·	·	·	·	
Type G	·	·	A	·	·	·	·	·	·	·	·	·	·	·	·	·	
Type H	**G**	**A**	**G**	**G**	**A**	**C**	**C**	**A**	**C**	**C**	**G**	**G**	**C**	**A**	**A**	**G**	(Consensus)
Type I	·	·	·	·	·	·	·	·	·	·	A	·	G	·	·	·	
Type J	·	·	·	A	·	·	·	·	·	·	A	·	G	·	·	·	
Type K	·	·	·	·	·	·	·	·	·	·	A	·	·	·	·	·	
Type L	·	·	·	·	·	·	·	·	·	T	·	·	·	·	·	·	
Type M	·	·	·	·	·	·	·	·	·	·	A	·	G	·	G	·	
Type N	·	·	·	·	·	·	·	·	·	G	·	·	·	·	·	·	
Type O	·	·	·	·	·	·	·	·	·	·	·	·	·	G	·	·	
Type P	·	·	A	·	·	·	·	·	·	·	·	·	A	·	G	·	
Type Q	·	·	A	·	·	·	·	·	G	·	·	·	A	·	·	·	
Type R	·	G	·	A	·	·	·	·	·	·	A	·	G	·	·	·	
Type S	·	·	A	·	·	·	·	·	·	·	A	·	G	·	·	·	
Type T	·	·	A	·	·	·	·	·	·	·	A	·	·	·	·	·	
Type U	·	·	A	·	·	·	·	·	·	·	A	·	A	·	·	·	

**Table 2:** DNA sequence characteristics of seven trios illustrating the mutational event. The standard paternity test, called a trio, involves the child, mother, and alleged father. Allele represents the number of 16 base pairs repeat units making up the entire allele. The alphabets in each sample represent the arrangement of the repeat types making up each allele. The alphabet code for the repeat types and the corresponding bases is given in Table 1. HinfI and Fnu4HI are the two restriction site polymorphisms located on the 5′ and 3′ flanking regions of the repeats, respectively, and are concordant between the progenitor and the mutated allele. The star (*) represents the mutated allele and aligned with the progenitor wherever possible. The progenitors are identified based on the sequence characteristic, SNP polymorphism, and the smallest allele difference between the parent and child. For the four indeterminate mutations, the mutated allele is aligned with both the possible progenitors.

		Sample	Allele	HinfI	Repeat types																															Fnu4HI	Comments		
1	Trio																																						

	1	*Mother-18 allele 1*	*18*	*Positive*	*A*	*B*	*C*	*D*	*D*	*E*	*C*	*H*	***H*** ^¶^	*I*	*I*	*H*	*I*	*J*	*I*	*I*	*L*	*G*														*Negative*	Possible progenitor	
	1	*Child-17 allele 1	*17*	*Positive*	*A*	*B*	*C*	*D*	*D*	*E*	*C*	*H*		*I*	*I*	*H*	*I*	*J*	*I*	*I*	*L*	*G*														*Negative*	**Deletion of one repeat unit**	
	1	*Father-18 allele 1*	*18*	*Positive*	*A*	*B*	*C*	*D*	*D*	*E*	*C*	*H*	***H*** ^¶^	*I*	*I*	*H*	*I*	*J*	*I*	*I*	*L*	*G*														*Negative*	Possible progenitor	
																																						
	1	Child-18 allele 2	18	Positive	A	B	C	D	D	E	C	H	H	I	I	H	I	J	I	I	L	G														*Negative*		
	1	Mother-18 allele 2	18	Positive	A	B	C	D	D	E	C	H	H	I	I	H	I	J	I	I	L	G														*Negative*		
	1	Father-22 allele 2	22	Positive	A	B	C	D	D	E	C	H	H	H	I	J	I	I	I	H	I	J	I	I	L	G										*Negative*		

2	Trio																																					

	3	*Father-18 allele 1*	*18*	*Positive*	*A*	*B*	*C*	*D*	*D*		*E*	*C*	*H*	*H*	*I*	*I*	*H*	*I*	*J*	*I*	*I*	*L*	*G*													*Negative*	Possible progenitor	
	3	*Child-19 allele 2	*19*	*Positive*	*A*	*B*	*C*	*D*	*D*	***D*** ^¶^	*E*	*C*	*H*	*H*	*I*	*I*	*H*	*I*	*J*	*I*	*I*	*L*	*G*													*Negative*	**Duplication of one repeated unit**	
	3	*Mother-18 allele 1*	*18*	*Positive*	*A*	*B*	*C*	*D*	*D*		*E*	*C*	*H*	*H*	*I*	*I*	*H*	*I*	*J*	*I*	*I*	*L*	*G*													*Negative*	Possible progenitor	
																																						
	3	Child-18 allele 1	18	Positive	A	B	C	D	D	E	C	H	H	I	I	H	I	J	I	I	L	G														*Negative*		
	3	Mother-25 allele 2	25	Negative	A	B	C	D	D	D	E	F	C	G	H	I	I	I	J	H	I	J	H	I	J	I	I	L	G							*Positive*		
	3	Father-31 allele 2	31	Positive	A	B	C	D	D	E	F	C	G	H	I	I	H	I	H	I	I	K	I	K	I	K	I	I	H	I	J	I	I	L	G	*Negative*		

3	Trio																																					

	7	*Child-19 allele 1	*19*	*Negative*	*A*	*B*	*C*	*D*	*E*	*F*	*C*	*G*	*H*	*I*	*I*		*J*	*H*	*I*	*J*	*I*	*I*	*L*	*G*												*Positive*	**Deletion of one repeated unit**	
	7	*Father-20 allele 1*	*20*	*Negative*	*A*	*B*	*C*	*D*	*E*	*F*	*C*	*G*	*H*	*I*	*I*	***I*** ^¶^	*J*	*H*	*I*	*J*	*I*	*I*	*L*	*G*												*Positive*	Progenitor	
																																						
	7	Child-24 allele 2	24	Negative	A	B	C	D	D	E	F	C	G	H	I	I	I	J	H	I	J	H	I	J	I	I	L	G								*Positive*		
	7	Mother-24 allele 1	24	Negative	A	B	C	D	D	E	F	C	G	H	I	I	I	J	H	I	J	H	I	J	I	I	L	G								*Positive*	Donor, child allele 2	
	7	Father-24 allele 2	24	Negative	A	B	C	D	D	E	F	C	G	H	I	I	I	J	H	I	J	H	I	J	I	I	L	G								*Positive*		
	7	Mother-31 allele 2	31	Positive	A	B	C	D	D	E	F	C	G	H	I	I	H	K	I	K	H	K	I	K	I	K	I	I	H	I	J	I	I	L	G	*Negative*		

4	Trio																																					

	8	*Child-21 allele 1	*21*	*Negative*	*A*	*B*	*C*	*D*	*E*	*F*	***F*** ^¶^	*C*	*G*	*H*	*I*	*I*	*I*	*J*	*H*	*I*	*J*	*I*	*I*	*L*	*G*											*Positive*	**Duplication of one repeated unit**	
	8	*Mother-20 allele 1*	*20*	*Negative*	*A*	*B*	*C*	*D*	*E*	*F*		*C*	*G*	*H*	*I*	*I*	*I*	*J*	*H*	*I*	*J*	*I*	*I*	*L*	*G*											*Positive*	Progenitor	
																																						
	8	Child-31 allele 2	31	Positive	A	B	C	D	D	E	F	C	G	H	I	I	H	I	H	I	I	K	I	K	I	K	I	I	H	I	J	I	I	L	G	*Negative*		
	8	Father-31 allele 2	31	Positive	A	B	C	D	D	E	F	C	G	H	I	I	H	I	H	I	I	K	I	K	I	K	I	I	H	I	J	I	I	L	G	*Negative*	Donor, child allele 2	
	8	Father-26 allele 1	26	Positive	A	B	C	D	D	E	F	C	G	H	I	I	H	I	H	I	I	I	I	L	S	J	I	I	L	G						*Negative*		
	8	Mother-31 allele 2	31	Positive	A	B	C	D	D	E	F	C	G	H	I	I	H	K	I	K	H	K	I	K	I	K	I	I	H	I	J	I	I	L	G	*Negative*		
																																						
5	Trio																																					

	10	*Mother-28 allele 2*	*28*	*Positive*	*A*	*B*	*C*	*D*	*D*	*E*	*C*	*G*	*H*	*I*	*K*	*K*	*I*	***I*** ^¶^	***H*** ^¶^	*I*	*I*	*H*	*I*	*J*	*I*	*H*	*I*	*J*	*I*	*I*	*L*	*G*				*Negative*	Possible progenitor	
	10	*Child-26 allele 1	*26*	*Positive*	*A*	*B*	*C*	*D*	*D*	*E*	*C*	*G*	*H*	*I*	*K*	*K*	*I*			*I*	*I*	*H*	*I*	*J*	*I*	*H*	*I*	*J*	*I*	*I*	*L*	*G*				*Negative*	**Deletion of two repeated units**	
	10	*Father-28 allele 1*	*28*	*Positive*	*A*	*B*	*C*	*D*	*D*	*E*	*C*	*G*	*H*	*I*	*K*	*K*	*I*	***I*** ^¶^	***H*** ^¶^	*I*	*I*	*H*	*I*	*J*	*I*	*H*	*I*	*J*	*I*	*I*	*L*	*G*				*Negative*	Possible progenitor	
																																						
	10	Child-28 allele 2	28	Positive	A	B	C	D	D	E	C	G	H	I	K	K	I	I	H	I	I	H	I	J	I	H	I	J	I	I	L	G				*Negative*		
	10	Mother-24 allele 1	24	Positive	A	B	C	D	D	E	C	G	H	I	K	K	I	I	K	I	I	H	I	J	I	I	L	G								*Negative*	Mother's sequence does not match child 26	
	10	Father-31 allele 2	31	Positive	A	B	C	D	D	E	F	C	G	H	I	I	H	K	I	K	H	K	I	K	I	K	I	I	H	I	J	I	I	L	G	*Negative*		

6	Trio																																					

	11	*Mother-24 allele 2*	*24*	*Negative*	*A*	*B*	*C*	*D*	*D*	*E*	*F*	*C*	*G*	***H*** ^¶^	*I*	*I*	*I*	*J*	*H*	*I*	*J*	*H*	*I*	*J*	*I*	*I*	*L*	*G*								*Positive*	Possible progenitor	
	11	*Child-23 allele 1	*23*	*Negative*	*A*	*B*	*C*	*D*	*D*	*E*	*F*	*C*	*G*		*I*	*I*	*I*	*J*	*H*	*I*	*J*	*H*	*I*	*J*	*I*	*I*	*L*	*G*								*Positive*	**Deletion of one repeated unit**	
	11	*Father-24 allele 1*	*24*	*Negative*	*A*	*B*	*C*	*D*	*D*	*E*	*F*	*C*	*G*	***H*** ^¶^	*I*	*I*	*I*	*J*	*H*	*I*	*J*	*H*	*I*	*J*	*I*	*I*	*L*	*G*								*Positive*	Possible progenitor	
																																						
	11	Child-24 allele 2	24	Negative	A	B	C	D	D	E	F	C	G	H	I	I	I	J	H	I	J	H	I	J	I	I	L	G								*Positive*		
	11	Mother-18 allele 1	18	Positive	A	B	C	D	D	E	C	H	H	I	I	H	I	J	I	I	L	G														*Negative*		
	11	Father-31 allele 2	31	Positive	A	B	C	D	D	E	F	C	G	H	I	I	H	K	I	K	H	K	I	K	I	K	I	I	H	I	J	I	I	L	G	*Negative*		

7	Trio																																					

	12	*Child-19 allele 1	*19*	*Positive*	*A*	*B*	*C*	*D*	*D*	*E*	*C*	*H*	*H*	*I*	*I*	*H*	*I*	*J*	***I*** ^¶^	*I*	*I*	*L*	*G*													*Negative*	**Duplication of one repeated unit**	
	12	*Father-18 allele 1*	*18*	*Positive*	*A*	*B*	*C*	*D*	*D*	*E*	*C*	*H*	*H*	*I*	*I*	*H*	*I*	*J*		*I*	*I*	*L*	*G*													*Negative*	Progenitor	
																																						
	12	Child-20 allele 2	20	Negative	A	B	C	D	E	F	C	G	H	I	I	I	J	H	I	J	I	I	L	G												*Positive*		
	12	Mother-20 allele 2	20	Negative	A	B	C	D	E	F	C	G	H	I	I	I	J	H	I	J	I	I	L	G												*Positive*	Donor, child allele 2	
	12	Mother-18 allele 1	18	Positive	A	B	C	D	D	E	C	H	H	I	I	H	I	J	I	I	L	G														*Negative*		
	12	Father-31 allele 2	31	Positive	A	B	C	D	D	E	F	C	G	H	I	I	H	I	H	I	I	K	I	K	I	K	I	I	H	I	J	I	I	L	G	*Negative*		

^¶^Represent the mutated region of the parental and child allele.
